# Mechanical Characterization of Pharmaceutical Powders by Nanoindentation and Correlation with Their Behavior during Grinding

**DOI:** 10.3390/pharmaceutics14061146

**Published:** 2022-05-27

**Authors:** Laura Baraldi, Davide De Angelis, Roberto Bosi, Roberto Pennini, Irene Bassanetti, Andrea Benassi, Guido Enrico Bellazzi

**Affiliations:** 1 Chiesi Farmaceutici S.p.A., Largo Belloli 11A, 43122 Parma, Italy; l.baraldi.consultant@chiesi.com (L.B.); d.deangelis@chiesi.com (D.D.A.); r.bosi@chiesi.com (R.B.); r.pennini@chiesi.com (R.P.); i.bassanetti@chiesi.com (I.B.); a.benassi@chiesi.com (A.B.); 2 Condensed Matter Theory Sector, International School for Advanced Studies (SISSA), Via Bonomea, 265, 34136 Trieste, Italy

**Keywords:** Nanoindentation, powder grinding, pharmaceutical powders, jet mill

## Abstract

Controlling the size of powder particles is pivotal in the design of many pharmaceutical forms and the related manufacturing processes and plants. One of the most common techniques for particle size reduction in the process industry is powder milling, whose efficiency relates to the mechanical properties of the powder particles themselves. In this work, we first characterize the elastic and plastic responses of different pharmaceutical powders by measuring their Young modulus, the hardness, and the brittleness index via nano-indentation. Subsequently, we analyze the behavior of those powder samples during comminution via jet mill in different process conditions. Finally, the correlation between the single particle mechanical properties and the milling process results is illustrated; the possibility to build a predictive model for powder grindability, based on nano-indentation data, is critically discussed.

## 1. Introduction

Characterizing single particle mechanical properties can be of paramount importance in the design of various manufacturing operations involving pharmaceutical powders or in the excipient selection during the early formulation of new drug products. To name just a few examples:

- To have a good chance of reaching the deep acinar airways, the aerodynamic size of the active pharmaceutical ingredient (API) particles in a dry powder for inhalation must lie between 1 and 5 μm [1,2]. This range of particle size is typically attained by milling a coarser, brittle starting material in a jet mill, where particle collisions and the consequent fragmentation are responsible for the particle size reduction [3,4,5]. The size reduction of API particles by milling is also frequently used to increase the dissolution, and thus the bio-availability, of poorly soluble drugs by enhancing the specific surface area accessible to the solvent [6,7,8];
- The ductility of the API and the excipient particles is very important during powder compaction; it determines the mechanical properties of the compact and thus the overall quality of the oral solid product (tablet) [9,10];
- Finally, a poor mechanical resistance of the composite particles, such as soft pellets or granulated particles, might lead to alterations in the drug product’s quality during conveying and handling operations [11].

Establishing a correlation between single particle mechanical properties and the behavior of powder products during manufacturing operations might promote novel effective design methods. Cheap and fast measurements on single particle mechanical properties could be used to exploit new targeted materials and to envisage innovative product solutions, thus significantly reducing the time and material consumption in trials at manufacturing plants.

Process design and control are nowadays increasingly assisted by numerical modelling and simulation [12,13,14]. For the realistic modeling of those processes where particle breakage occurs, both as a desired [15,16,17] or a detrimental effect [18], a breakage probability distribution for the mother particle and a fragmentation distribution describing the generation of daughter fragments must be properly defined [19]. Meier et al. demonstrated how the parameters appearing in such functions directly depend on the single particle mechanical properties [20,21].

**Figure 1:**
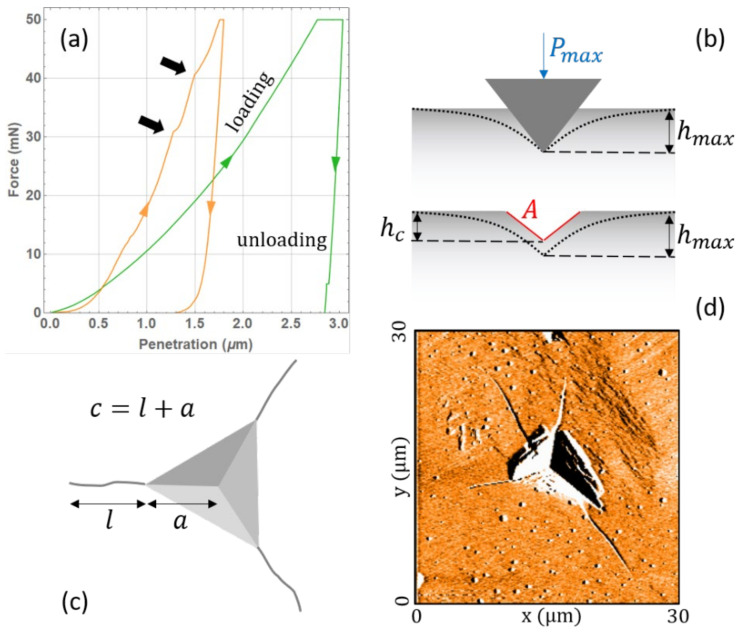
(**a**) Mechanical hysteresis curves for sodium chloride (green) and lactose (yellow); black arrows indicate step-like features in the ascendant part of the loop due to crack propagation; (**b**) sketch of tip penetration during indentation and footprint depth; (**c**) sketch of the top view of a footprint with crack length measure; (**d**) AFM image of an indentation footprint on a compound A particle.

The nano-indentation technique enables the measurement of single particle mechanical properties with practically no consumption of material, e.g., only a few tens of particles are necessary. Such versatility is particularly appreciated when dealing with an API of a new synthesis, whose total amount, in the early development phases, is limited, while the synthesis costs are considerable. A typical nano-indentation test consists of pushing a hard metallic tip on a single powder particle, glued onto a sample holder, with increasing force, while measuring the tip displacement [22,23,24]. The typical indentation profiles acquired on our samples are shown in Figure 1a. Part of the tip displacement is due to the elastic deformation of the particle surface and part is due to plastic penetration inside it, as illustrated in Figure 1b. From the recorded curve, it is thus possible to extract information on both the elastic response and the plastic (irreversible) deformation. By imaging the indentation footprint with optical microscopy, atomic force microscopy (AFM), or scanning electron microscopy (SEM), it is possible to measure the length of the generated cracks. Examples are shown in panels (c) and (d) of Figure 1. Assuming a certain model of deformation and crack propagation beneath the tip, a brittleness index can be defined indicating the nature of the plastic deformation, i.e., whether the material responds in a brittle or a ductile fashion.

Naturally, single particle nano-indentation also has some intrinsic limitations and is generally less controllable and reproducible than its counterpart on tablets and powder compacts [10,22,25]. Powder particles must be tightly glued to a sample holder to be manipulated and placed individually under the indenter. This is only possible if the particles have a size of at least 50–100 μm; below such a limit, cohesion forces will dominate over gravity, keeping the powder particles aggregated. Powders with a peculiar crystal habit (e.g., acicular, lamellar) will lie on the sample holder with certain preferential orientations, i.e., certain crystallographic directions might not be accessible to indentation. In other cases, the single crystal shape (e.g., octahedra) might prevent the glued particles from exposing horizontal surfaces, i.e., the planes parallel to the sample holder and perpendicular to the indentation axis; if this alignment condition is not met, the indentation data are meaningless. If the indentation footprint is too small, a local or “microscopic” value of the elasto-plastic properties of the material is probed; thus, a large variability in the measures must be expected due to surface inhomogeneities, local disorder, and defects. Only if the indentation footprint is large enough to average over the microscopic surface disorder and to probe enough “weak spots” of the particle surface will the data variability be substantially reduced, and the results will no longer depend on the indentation depth (meaning also the maximum indentation force and footprint size) [24].

Several works are present in the literature discussing the application of single particle nano-indentation to active pharmaceutical ingredients and excipients [26,27,28] and its correlation with powder processability [20,22,29,30,31]. Shariare et al. [27] demonstrated the difficulty in correlating directly the brittleness properties of pharmaceutical powders with the calculated interaction energy between crystalline planes within the particles. This difficulty stems from the fact that the material breakage is dominated by defects, dislocations, and pre-existing flaws, which might considerably lower the theoretical energy barrier to initiate slip motion between the molecular planes, as proved by Vegt et al. [31,32]. Zuegner et al. [30] found that the connection between powder milling efficiency and the elastic and plastic properties of single particles is non-trivial. They concluded that particle elasticity impacts the breakage resistance of materials more than particle hardness. However, their picture cannot explain the behavior of materials such as sodium chloride, and without any further measurement, it is not possible to determine whether the plastic deformation of the particles has a ductile or a brittle character. This problem was further addressed by Meier et al. [20], who showed how the brittleness index remains the most promising parameter to predict the milling behavior of powders. Taylor at al. [29] found a very nice correlation between the milling efficiency and different single particle mechanical properties, such as the Young modulus, the hardness, and the brittleness index of different pharmaceutical compounds. The single particle nano-indentation data also correlate very well with the powder behavior during the compaction and tableting processes; a summary can be found in the review by Egart et al. [22].

In this work, we characterize the elastic and plastic responses of different pharmaceutical powders by measuring the Young modulus and hardness via nano-indentation. The estimation of the average length of the generated cracks allows the evaluation of the fracture toughness and brittleness index. Our results are compared with those already present in the literature; the variability and reproducibility of the measurements are also analyzed. Particle comminution is then studied through a tabletop jet mill, and the size reduction effects are discussed for the different materials and process conditions. Finally, the correlation between the single particle mechanical properties and the powder grindability is illustrated and discussed, as is the possibility of building a predictive model for the powder behavior during milling, solely based on the single particle mechanical properties measured via nano-indentation.

## 2. Materials and Methods

### 2.1. Materials Selection

The APIs and excipients for the nano-indentation analysis were selected based on three different criteria. First, our choice was limited by the sample abundance as we needed a few tens of grams of powder to perform the jet-milling trials. Such quantities are not easily granted for new compounds whose synthesis reaction is still in its early phase of design. A second important aspect is that, to validate our indentation method, we needed a reference compound whose mechanical properties were already known and whose crystalline nature was similar to those materials we synthesize in-house. Commercial excipients were selected for this purpose: sodium chloride and L-(+)-tartaric acid from Merck^®^ (Darmstadt, Germany) and lactose α-monohydrate from Armor-Pharma^®^ (Loudeac, France). The sodium chloride and lactose were both analyzed as they appeared out of the box and were also re-crystallized by slow evaporation from a saturated water (sodium chloride) or ethanol (lactose) solution. Finally, our material selection was also driven by the availability of suitably sized and shaped crystalline particles, large enough to be manipulated and glued while exposing large and flat surfaces. Different particle/crystal morphologies were explored, from irregular prisms, passing through large plates, to elongated homogeneous needles. While crystals of compound A were directly used as obtained by bulk synthesis, for compounds B and C a re-crystallization procedure was necessary to achieve larger particle sizes. For the single crystals of compounds A and B, the largest particles were selected and inspected at the stereo microscope, while for the bulk crystals of compound C a gentle sieving step with a 125 μm mesh was necessary, before particle selection, in order to separate a pre-existing fine particle fraction.

### 2.2. Particle Size Characterization

The particle size distribution (PSD) of the starting and micronized material was determined using a Sympatec HELOS/BF instrument equipped with a RODOS/ASPIROS dry dispersion unit. The Sympatec data are presented in terms of equivalent volume density distribution q3lg. The powder dispersion was performed at a 2-bar pressure drop with a sled speed of 30 mm/s. Different combinations of R1 (0.1/0.18–35 μm), R3 (0.5/0.9–175 μm), and R5 (0.5/4.5–875 μm) lenses allowed the covering of the entire particle distribution range for the samples. The PSD characterization of the micronized material was always performed within a few hours of the milling; all the samples were stored in a dry nitrogen atmosphere to avoid changes in their surface characteristics possibly leading to particle sintering. The presented PSD and *dv*90 (i.e., the particle diameter corresponding to the 90th percentile of the volume equivalent particle size distribution [33]) data were averaged over three replicas of the analysis, with an estimated uncertainty below 1 μm.

### 2.3. Indentation Test

#### 2.3.1. Sample Preparation

**Figure 2:**
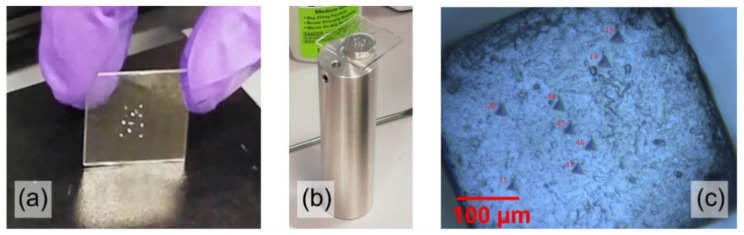
(**a**) An example of glass slide with selected particles glued on it; (**b**) the glass slide mounted on the sample holder apparatus before the insertion in the indentation equipment; (**c**) some indentation footprints imaged by optical microscopy on a sodium chloride particle.

For each measurement set, the particles were carefully selected using stereo and optical microscopy in order to find and isolate those which were sufficiently large, with smooth and uniform surfaces. Precision tweezers were employed to glue the selected particles on a dedicated square glass slide where a thin film (<500 μm) of biphasic epoxy glue had previously been deposited. The particles were deposited in the central part of the glass slider only, as shown in Figure 2a. After 15 min, the glass slide was very gently brushed to remove powder residues, dust agglomerates, or particles not perfectly glued. The glass slide was in turn glued on the nano-indenter sample holder and left drying for at least 30 min; see Figure 2b. It can be noted that all the glued particles lie within the metallic cylinder area of the sample holder: indenting too far from it would add a spurious elastic response due to the glass slide bending. In Figure 2c, an optical microscopy image shows several indentation footprints on the same sodium chloride particle.

#### 2.3.2. Indentation Method

**Figure 3:**
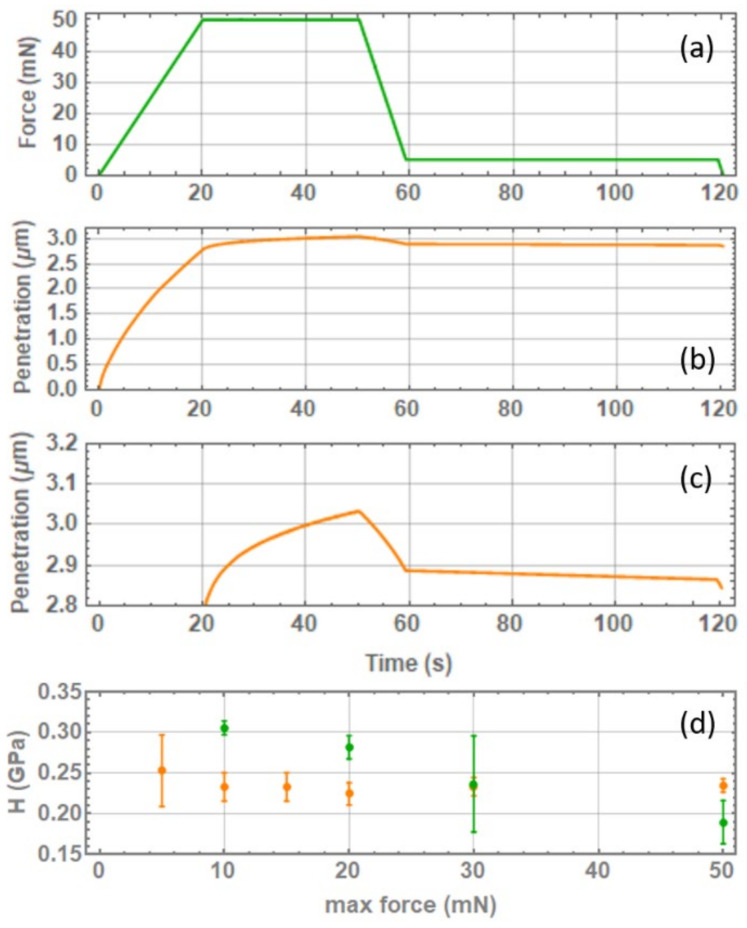
(**a**) Applied force as a function of time during a typical indentation cycle; (**b**,**c**) penetration depth h as a function of time when the force profile of panel (**a**) is applied on a sodium chloride particle; (**d**) some examples of how the measured mechanical properties, the hardness H in this specific case, depend on the maximum loading force $P_{max}$ for re-crystalized sodium chloride (yellow) and compound c (green).

The indentation experiments were performed using a NanoTest™ Vantage nano-indenter (from Micro Materials^®^, Ltd., Wrexham, UK) equipped with a sharp pyramidal Berkovich diamond indenter. The typical indentation protocol is illustrated in Figure 3a; it consists of a loading ramp of 20 s duration, a 30 s dwell period at maximum load $P_{max}$, and a final unloading step lasting 10 s. The corresponding sample deformation profile is shown in Figure 3b,c. Notice how the tip keeps penetrating the sample even when the loading force is kept constant; this occurs due to creep deformation. The purpose of the dwell period at maximum loading is to let the creep rate reduce so that the plastic deformation is minimized, or completely absent, during the subsequent unloading step, in which the elastic character of the sample is probed. At the very end of the unloading curve, with only 10% of $P_{max}$ still applied, a second dwell period of 60 s is maintained to estimate the tip thermal drift and correct for this effect in the Young modulus E and hardness H  calculation. For the different materials and samples, the maximum applied load ranged from 5 to 200 mN depending on the ease in the crack production without the generation of major damages to the particle, i.e., chipping or particle breakage.

#### 2.3.3. Elasto-Plastic Constants Determination

The indentation hardness H is a measurement of the attitude of a material towards being irreversibly deformed by an external mechanical stress, exerted through a tip of known shape. With reference to Figure 1b, H is defined as:

(1) $$H=\frac{P_{max}}{A\left(h_{C}\right)},$$

where $P_{max}$ is the maximum force applied by the indenter tip on the sample surface, and A is the area of the generated indentation footprint. The latter is a function of the tip shape and penetration depth $h_{C}$; for the Berkovich tip used in this work, the correlation between the footprint area and the penetration depth is given by $A=24.56\;h_{c}^{2}$. Notice how, as an irreversible deformation of the sample surface occurs, the loading and unloading curves do not coincide, i.e., a mechanical hysteresis loop is recorded. The reason for using $h_{C}$ in the hardness calculation rather than $h_{max}$ is that the latter contains both the plastic and the elastic deformation, as sketched in Figure 1b.

From the slope of the unloading curve, it is also possible to infer the Young modulus value E, representing how prone a material is to elastic deformation under the action of an external force:

(2) $$E=\frac{\sqrt{\pi }}{2\beta }\frac{dP}{dh}\frac{1}{\sqrt{A\left(h_{C}\right)}},$$

where P is the pressure exerted on the indenter tip, h is the penetration depth, and β is a tip geometry constant (β=1.034 for our Berkovich indenter [22]). As the recorded curves usually present some noise, the dP/dh derivative cannot be easily computed numerically via finite-difference algorithms; rather, the instrument software usually fits the unloading curve with some model expression of a known analytical derivative [34]. Under the same external force, a material with a small E value will show a significant reversible deformation, e.g., rubber; a material with a large E will give rise to an infinitesimal elastic deformation, e.g., steel. Notice that the E appearing in Equation (2) is the reduced Young modulus:

(3) $$\frac{1}{E}=\sum_{i}\frac{\left(1-\nu_{i}^{2}\right)}{E_{i}},$$

where $E_{i}$ and $\nu_{i}$ are the Young modulus and the Poisson ratio of each deformable medium (in series) in the measurement system, i.e., the tip, the powder particle, the glue layer, and the sample holder. E coincides with the particle young modulus $E_{p}$, only assuming that the tip, the glue layer, and the sample holder are orders of magnitude stiffer than the particle and thus remain practically undeformed during the whole indentation process. Only in this specific situation:

(4) $$\frac{1}{E}≅\frac{\left(1-\nu_{p}^{2}\right)}{E_{p}}.\;$$

As $\nu_{p}$ is very difficult to measure on single particles, the value of E is usually reported in the literature rather than $E_{p}$ [24]; we have adopted the same convention throughout the rest of the paper. In any case, the typical values of $\nu_{p}$ for the crystals of the organic molecules are 0.2–0.25; for sodium chloride, $\nu_{p}=0.18$ [31], which means $E_{p}∼0.97\;E$, i.e., the difference between the Young modulus and the reduced modulus is smaller than the typical uncertainty in the measurement itself.

At least two independent measurement campaigns were performed for each selected material, employing different operators and with independent sample preparation to evaluate the method’s reproducibility and to test its sensitivity to sample preparation. For each $P_{max}$ value, 5 to 10 indentation tests were performed over 2 or more particles. For some of the investigated materials, we noticed the typical overestimation of E and H, as well as their larger variability when small $P_{max}$ values were applied. An example is shown for sodium chloride in Figure 3d. For the other materials, we notice that both the E and the H values keep decreasing slightly with the increasing $P_{max}$ and that the variability of the measurement is not necessarily correlated to $P_{max}$; see the compound C curve in the same figure. Increasing $P_{max}$ further results in particle chipping or breakage, thus preventing us from reaching a condition where E and H are completely independent from $P_{max}$. For this reason, we decided to present our measurements as an average over all of the applied $P_{max}$ range.

### 2.4. Footprint Analysis

#### 2.4.1. Imaging Methods

By inspecting the indentation footprint, it is possible to assess the nature of the irreversible deformation, i.e., whether the mechanical energy lost during the irreversible deformation is dissipated in a ductile displacement of the material around the footprint or through brittle fractures. The fracture toughness $K_{C}$ is a critical stress above which the cracks, naturally present in a particle, start to propagate irreversibly, thus breaking it into smaller fragments. Such a value is calculated by combining the previously defined mechanical properties H and E with the crack length l or the other characteristic length c defined in Figure 1c, which is measured through optical or atomic force microscopy. The crack footprints were investigated by optical microscopy (Nikon^®^ Eclipse LV 100POL, Tokyo, Japan) and by atomic force microscopy (C3000 by Nanosurf^®^AG, Liestal, Switzerland). The AFM probe was operated using the imaging mode and in the dynamic force acquisition/tapping mode with a long cantilever 190AI-G. The Z-controller setpoint, the P/I gain, the tip voltage, and the free vibration amplitude were modified during the analysis depending on the sample nature in order to produce the most resolved image possible.

#### 2.4.2. Fracture-Related Constants Determination

Several models have been proposed for $K_{C}$ based on different assumptions about the deformation and crack propagation beneath the tip [35]. Meier et al. tested 19 of them on sucrose, finding up to one order of magnitude variability in the results [20]. In this work, we limit our analysis to the most commonly employed model of Laugier:

(5) $$K_{C}=\gamma \;\sqrt{\frac{E}{H}}\frac{\;P_{max}}{c^{3/2}},$$

where γ = 0.016. Now, a brittleness index b can be defined as:

(6) $$b=\frac{H}{K_{C}},$$

such a ratio is very small either when the material is hardly plastically deformable (small H) or when the stress necessary to start the crack propagation is very large ($K_{C}$ large); in both cases, the material will not deform at all, or it will undergo a ductile deformation. Conversely, a large b value is associated with small stresses to initiate crack propagation (small $K_{C}$) and thus to particle breakage by brittle fracture. Another interesting quantity to estimate, once $K_{C}$ is known, is the critical diameter $d_{c}$, i.e., the characteristic particle size below which brittle fracture no longer occurs [36,37,38]. If fragmentation by brittle fracture was the only size reduction mechanism, $d_{c}$ could be a measure of the smallest particle size attainable with any milling process. In fact, particles with a diameter smaller than $d_{c}$ could only deform in a ductile manner under compressing loads. Kendall [39] and Hagen [40] independently proposed two different models for this ductile–brittle transition, obtaining for $d_{c}$ the same dependence on $K_{C}/H$:

(7) $$d_{c}=\alpha {\left(\frac{K_{C}}{H}\right)}^{2},$$

in this work, we adopt the Hagen model, where α=29.5; more details about the different model assumptions can be found in the work of Knieke [37].

Crack imaging and analysis is a time-consuming operation; we thus performed it only on a limited subset of the indentation sessions, at least one for each selected material. If not otherwise stated, the presented results are the average over at least five different footprints.

### 2.5. Micronization

Size reduction was performed through a LaboMill jet mill from Food Pharma Systems^®^ (Fiorenzuola, Italy), equipped with a 1.5-inch PTFE milling chamber and 4 inert stainless-steel nozzles. The mill was fed with 1.6 g of powder manually dosed regularly in time to maintain a constant feed rate of 10 g/h. Nitrogen was selected as the milling gas. All the materials were tested at different grinding pressures, namely 3, 4.5, and 6 barg. The feeding/injection pressure was set 1 barg above the grinding one to avoid blow-back phenomena.

## 3. Results and Discussion

The data on the elastic and plastic behavior acquired for all the samples are summarized in Table 1; the values present in the literature for the reference compounds are also reported for comparison (sodium chloride [26,30], tartaric acid [20], and lactose [20,30]). The hardness of the sodium chloride is to be found in good agreement with the data reported by Duncan-Hewitt et al. [26], while some discrepancy is found for the Young modulus. This material is known to be extremely ductile with a very limited elastic deformation, which makes the unloading curve of the hysteresis loop very steep and thus extremely hard to be correctly fitted for the extrapolation of its derivative by the instrument software. Testing different fitting formula and different numerical methods to determine the fitting coefficients, through an external software, we found that E can increase even by 5–7 GPa. This brings the re-crystallized sample value into agreement with the experimental one and slightly enhances the commercial sample value, which remains lower. Though with a little more variability between the different sessions, the hardness of the tartaric acid is also close to the value reported by other authors; the Young modulus is found to be in good agreement except for the commercial session 2, where again we recorded an underestimation. Drops in the E value could be justified by particle gluing issues. If the glue layer is not perfectly stiff, and it undergoes a significant deformation, two terms of Equation (3) must be retained, leading to $E>E_{p}$; in particular, if the deformation of the glue layer is comparable to the particle one, we obtain $E∼E_{p}/2$. Both E and H of lactose are in reasonable agreement with the data reported in the literature; a slightly higher variability is visible for H among the different sessions.

For sodium chloride and lactose, it is possible to assess the effect of re-crystallization on the measured mechanical properties. For both materials, the Young modulus and hardness are found to be larger than for the out-of-the-box counterpart. We are thus led to the reasonable conclusion that a small-scale, controlled re-crystallization makes the powder particles harder and more elastic. This finding is in agreement with the results published by de Vegt et al. [31], showing how an increasing density of pre-existing flaws lowers both E and H. On the other hand, on a large commercial scale, reaching the crystal perfection is far from trivial: in the crystal packing, flaws and defects compromise the hardness. The contained variability among the different measurement sessions for compounds B and C reveals a good reproducibility of the gluing procedure and a weak dependence on the operator. The same is true for sessions 3 to 5 of compound A, all performed on the same batch, while a pronounced difference is visible when compared with tests 1 and 2 performed on two other different batches, even though they are obtained from similar synthesis processes. This finding highlights the importance of characterizing the mechanical properties of every single manufactured batch before setting the milling process specifics and, in any case, it warns about the intrinsic batch-to-batch variability that, for certain molecules of new synthesis, can be significant.

The crack length *c* was determined only on a subset of the measurements presented in Table 1; $K_{C}$, b, and $d_{C}$ were subsequently calculated. The results are summarized in Table 2. Sodium chloride particles are extremely ductile and, even applying a high maximum loading $P_{max}=500\;\mathrm{mN}$, we never succeeded in inducing cracks around the indentation footprints. This behavior has also been discussed by other authors, with the exception of Duncan-Hewitt et al. [26], obtaining the values reported in Table 2. Indeed, their $K_{C}$ value is very high compared to all the other materials analyzed here, confirming no attitude to brittle fracture, i.e., b→0. For tartaric acid, our $K_{C}$ and b values compare nicely with the literature data, while the lactose results are in good agreement with what concerns $K_{C}$ and reveal a smaller value for b. As already noted for E and H, compound A shows a considerable batch-to-batch variability; for session 3, only one indentation produced good cracks, and thus, no standard deviation is reported. Moreover, the three sessions on compound B give consistent and reproducible measurements. Compounds B and C appear to be the most brittle; they are thus expected to be easily grindable in milling processes.

The standard deviations associated with all of our measurements are higher than those reported by the other authors for the commercial excipients. This is in part due to our choice of averaging over all the applied loads, including the lowest loads, which typically enhanced the measurement variability. A deep investigation of how the indentation protocol might affect the results variability is certainly necessary. In particular, the impact of the loading and unloading rates, as well as the importance of the dwell time, must be assessed. This point is poorly discussed in the literature, and as it would require a large measurement campaign, focusing on few materials, this is beyond the scope of the present paper.

**Figure 4:**
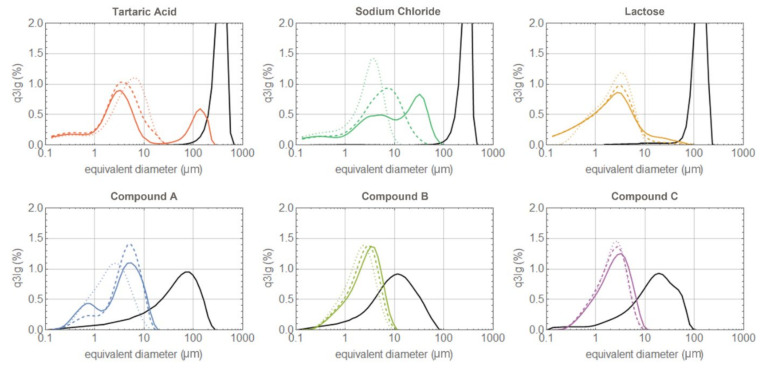
PSD of the different materials before and after grinding. The black lines represent the starting material PSD; the colored continuous, dashed, and dotted lines represent the ground product PSD at 3, 4.5, and 6 barg grinding pressure, respectively.

**Figure 5:**
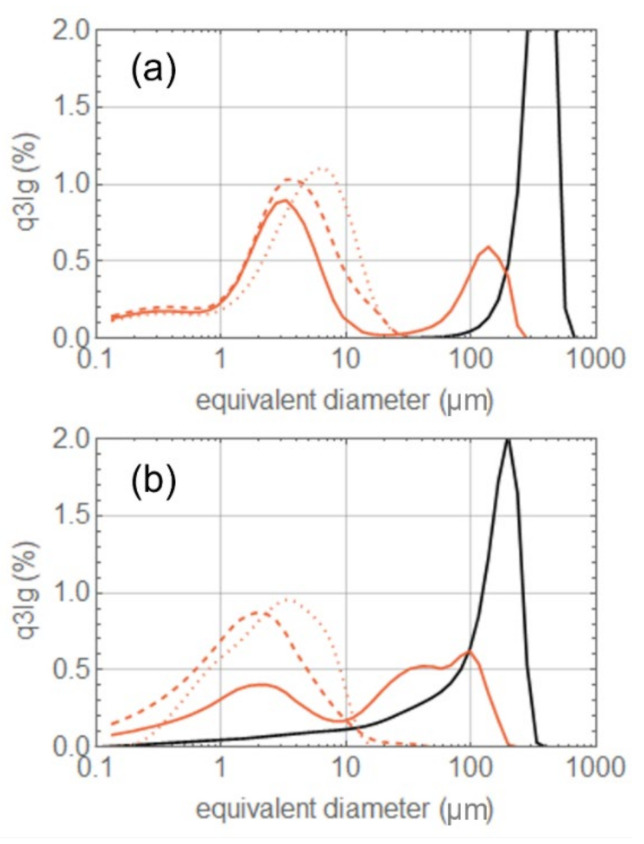
PSD of tartaric acid for different grinding pressures with a coarser (**a**) or finer (**b**) starting material. The black lines represent the starting material PSD; the red continuous, dashed, and dotted lines represent the ground product PSD at 3, 4.5, and 6 barg grinding pressure, respectively.

Both the synthesized compounds and the commercial excipients (in their out-of-the-box form) were milled at the same powder feed rates and grinding pressures. The PSD of the processed material was acquired immediately after milling; the results are summarized in Figure 4. Compound C is indeed the most easily grindable; at 3 barg, its PSD is already close to the limiting distribution with a *dv*90 well below 10 μm. Further increases in the grinding pressure only slightly sharpen it, without any significant left shift. The same applies for compound B, with only a minor shift observed with the increasing pressure. Compound A is harder to mill; at low pressure, the collisions are not especially effective in reducing the particle diameter, which results in high particle residence time, i.e., a large hold-up mass accumulates in the mill chamber, reducing even further both the nitrogen and the particle velocity [41]. At small collision velocities, chipping and abrasion mechanisms are known to begin, generating small size fragments which could lead to the observed bi-modal size distribution [42,43,44]. Another possible scenario justifying a bi-modal distribution at low grinding pressure is an intermittent grinding regime [41]. If the product is difficult to grind, then, as the particles accumulate in the milling chamber, the large hold-up mass slows down the fluid and its centrifugal force until most of the particles are able to reach the classifier and escape the mill, even though still large in size, giving rise to the rightmost peak. When the mill is almost empty, the fluid accelerates, promoting high energy collisions which are very effective in size reduction and preventing the escape of large particles by restoring a stronger centrifugal force. In this way, only small size particles escape the mill, giving rise to the leftmost peak; this optimal milling condition is gradually lost as the milling chamber refills again. Only at 6 barg are the collisions effective in provoking brittle fracture. This restores a mono-modal PSD slightly left-shifted. Unfortunately, the milling equipment does not allow for a further increase in pressure to verify whether such a distribution is already the limiting one. Something similar happens for lactose, but here, it is clear the limiting distribution is already reached at 4.5 barg. Sodium chloride also displays a bi-modal distribution at low pressure, which disappears with increasing pressure as the PSD shifts to the left and sharpens. The mill is clearly also working in an intermittent regime for tartaric acid, and during its periodic emptying, the particles exit with almost the same size they entered it with. Increasing the grinding pressure causes the intermittent regime to disappear, but the main peak shifts to the right. This can be due to two phenomena: with higher collision speed, chipping and abrasion mechanisms are suppressed and brittle fracture produces only larger fragments; alternatively, a regime where milling efficiency decreases with increasing grinding pressure is known to occur at high feed rates [41]. The milling pressure should be increased even more to see the PSD peak shifting back to the left. One might argue that the increasing difficulty in grinding certain materials (sodium chloride and tartaric acid in particular) is due to the larger particle size of the feed rather than to a less brittle attitude. To demonstrate that this is not a correct interpretation, we sieved the tartaric acid material, removing the coarser fraction from the feed. The difference between the original feed and the sieved one can be appreciated by comparing panels (a) and (b) of Figure 5. Probably due to the fracture of some weak large particles, the new feed shows a left tail enhancing the content of particles with a diameter between 10 and 100 μm; the overall feed PSD closely resembles the lactose one. However, the milled PSD and the powder behavior, with increasing grinding pressure, remain identical to the coarser feed.

Reducing the effect of the hold-up and accessing higher grinding pressures, to also reach the limit PSD for hard materials, could both be achieved using a larger mill. However, working with a larger grinding chamber implies the use of bigger powder samples, requiring tens of grams instead of a few grams per trial, and such an amount of powder is generally not available in the early stages of new drug synthesis.

In setting the milling process parameter for a new material, we are primarily interested in:

(i) Understanding whether the particle size can be reduced below a certain value;
(ii) How high the grinding pressure must be raised to achieve the desired PSD. As this depends also on the powder feed rate, we should consider the specific milling energy; however, all the presented results were collected at the same feed rate; thus, the specific milling energy varies with pressure only;
(iii) How strong the PSD of the milled material depends on our choice of milling pressure;
(iv) Estimating the smallest particle size attainable, as this determines the span of our PSD.

**Figure 6:**
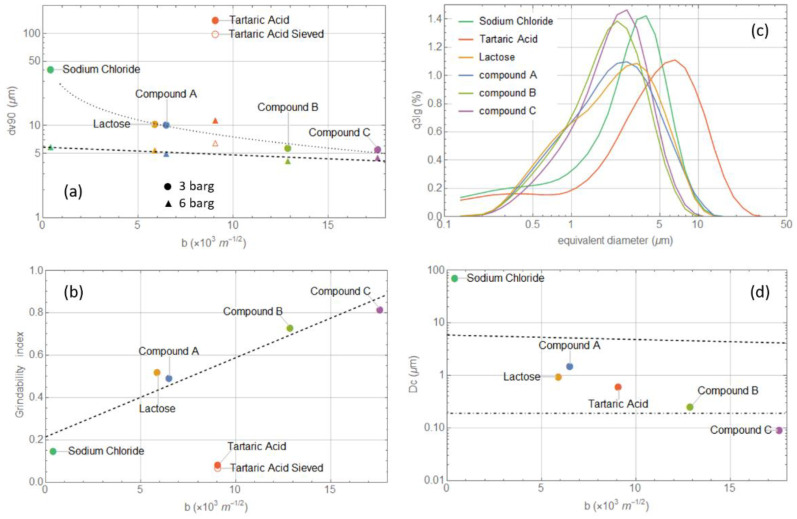
(**a**) *dv*90 as a function of the brittleness index b for 3 and 6 barg milling pressure (circles and triangles, respectively); the black dashed and dotted lines represent the fit of Equations (8) and (9); (**b**) grindability index as a function of the brittleness index b; the black dashed line represents the fit with Equation (10); (**c**) overlay of the PSD of the different samples milled at 6 barg grinding pressure; (**d**) critical particle diameter estimated through Equation (7) (colored dots); average diameter of the smallest fragments as measured from the PSD of panel (**c**) (dot-dashed line) and Equation (8) fit (dashed line, the same as panel (**a**)).

To investigate point (i), the dv90 of the milled samples was plotted against their brittleness index for different grinding pressures; see Figure 6a. Given the impossibility of generating cracks on sodium chloride, we used the b value from Duncan-Hewitt et al. [26]; for compound B, we averaged b over the data from the different sessions; for compound A, where a large batch-to-batch variability is present, we used only the b value measured directly on the milled batch. With the exception of tartaric acid, whose strongly bi-modal distribution makes the dv90 of little use, there is indeed a correlation. The three barg data appear more scattered as the low-pressure PSD retains memory of the starting material one; at 6 barg, most of the materials reach the limiting distribution and the differences in dv90 become narrow. By fitting the data, it is possible to estimate a dependence of dv90 on the measured brittleness index b; for the 3 and 6 barg data, we get, respectively:

(8) $$dv90=5.8\;e^{-b/51.6},$$

(9) $$dv90=30.9/b^{0.43}-4.0,$$

whose predictivity is of course valid only for a certain milling pressure and our specific mill geometry. More general expressions with an explicit dependence on pressure could be defined collecting data on many more materials and at different pressures; they could be used to answer to point (ii), i.e., to predict the dv90 of a new material just by estimating its b via nano-indentation. Looking at panel (a) of Figure 6, it is also clear how the difference in dv90 between 3 and 6 barg becomes narrower as b increases. Very brittle materials should in fact already reach their limiting distribution at very low pressures, while harder materials lose memory of their starting material PSD only at very high pressure. Thus, similarly to what the other authors have done, we propose the definition of an index representing the ease of milling a powder, a form of grindability index, as $g=(dv90_{3barg}-dv90_{6barg}$)/$dv90_{3barg}$. Considering that this index is estimated by comparing the PSD of two milled samples rather than by referring to the starting material PSD, this index is by construction suitable to answer to point (iii). The grindability index g, as a function of b, is plotted in panel (b) of Figure 6. Again, with the exception of tartaric acid, all the points lie on the following fitting curve in Equation (10).

(10) g=0.037 b+0.21

which again has a predictivity limited to a certain pressure range and bound to our specific milling equipment. Why tartaric acid does not follow the general trends highlighted here for the other materials remains unclear. According to its b value, it should be a medium brittleness material; however, its behavior in the mill suggests an extreme difficulty in size reduction, with the accumulation of a large hold-up mass. Its large Young modulus, associated with large feed particles, might completely change the particle dynamics inside the mill: random frequent elastic rebound significantly lowers the average particle-particle and particle-wall velocity, reducing the comminution efficiency by brittle fracture. However, these mechanisms should also be at work for sodium chloride; it also has a similarly large E and even less attitude to brittle fracture, and still, it is found to be more easily grindable than tartaric acid.

Panel (c) of Figure 6 shows the overlay of the PSD for all the samples milled at 6 barg; assuming these distributions closely resemble the limiting ones (which is verified only by compounds B and C and for lactose), one can verify the predictivity of the Hagen model and try to answer point (iv). For compounds A, B, and C, as well as for lactose, the smallest fragment size is around 0.2 μm; for sodium chloride and tartaric acid, longer left tails suggest the presence of much smaller fragments. There is however a spurious numerical effect emerging when combining the measurements of different lenses to generate a unique PSD. Checking the PSD using the R1 lens, we also found for sodium chloride and tartaric acid a minimum fragment size of 0.2 μm. The smallest fragment size attainable by brittle fracture, as predicted by the Hagen theory, is calculated through Equation (7), reported in Table 2, and plotted in Figure 6d. The predicted and measured data are in good agreement only for lactose and compound B. The prediction for compound A, tartaric acid, and sodium chloride significantly overestimates the smallest attainable particle size by orders of magnitude; an underestimation occurs for compound C. We must conclude that, for these compounds, either the simple assumptions underlying the Hagen model are not correct or brittle fracture is not the sole size reduction mechanism at play. The inadequacy of the Hagen model has also been reported by other authors showing how single particles can break into fragments much smaller than the calculated $d_{c}$ [37]. Their argument is that the simple models for the estimation of $d_{c}$ are based on static stress distributions in the samples and are thus more suitable for quasi-static loading experiments, rather than for high-speed collisions such as those occurring in single impaction testers (or jet-impact testers), as well as in jet mills. Further investigations in this direction would require dedicated work and the use of a particle size analysis tool more suitable for the sub-micron range, i.e., the application of Mie light scattering theory with the wet dispersion laser diffraction method.

## 4. Conclusions

Once the proper sample preparation procedure had been set up, the indentation analysis proved to be reliable and reproducible. The measurements on commercial excipients were in good agreement with the data available in the literature. However, the single particle handling and gluing, the search for good and flat terraces for indentation, and the measurement of the crack length are all manually executed operations which make nano-indentation a very time-consuming analysis. Another main weakness of the technique is the need for re-crystallization to generate larger particles for those compounds whose selected synthesis path leads to the precipitation of very small crystals. This is not only another time-consuming procedure, but it might significantly alter the mechanical properties of the particles, making them not representative of the larger amount of powder used in the milling trials. The differences in the mechanical properties of re-crystallized and out-of-the-box commercial excipients are evident from our analysis; however, re-crystallization did not prevent us from obtaining a good correlation between dv90 and b for compounds B and C. This might mean that, for these compounds, the re-crystallization process does not significantly alter the measured mechanical properties. Indeed, the industrial-scale synthesis process of the commercial excipients, and their post-synthesis handling, differs significantly from the lab-scale synthesis of compounds A, B, and C. It is thus reasonable to expect for them a more pronounced impact of re-crystallization. Finally, it must be noted that the nano-indentation technique alone is not able to give any prediction about the possible alterations to the solid-state properties of the milled material as a consequence of the received mechanical stress. This is a very important aspect to consider while designing a milling process, and to date, it is possible to evaluate it only on the milled samples, i.e., on a trial-and-error basis. For all the above-mentioned reasons, we believe nano-indentation remains of great value only in the very early development phases, when new compounds are synthesized in the order of a few grams. With such limited abundance, milling process design cannot be afforded on a trial-and-error basis. However, when the synthesis scale is many tens of grams, a few milling trials (guided by the data collected in the past on other compounds) should allow the setup of a decent milling process in a shorter time compared to the duration of an indentation analysis.

Despite all the limitations of nano-indentation, a correlation exists between the brittleness index b and the powder behavior during milling, and it can certainly be exploited to build predictive models. The dependence of the dv90 or the grindability index upon b deserves further investigation, enriching the statistics with new compounds, especially those with little brittle fracture attitude. Another aspect to better clarify is how strongly the dv90 and the grindability index still depend on the particle size of the feed material. The classification mechanism of jet mills should eliminate by design the dependence of the milled product PSD on the starting material one; however, this is true only in an ideal working regime, which might not be met here for small b compounds. With a much larger set of milling experiments, it is certainly possible to define a generalized version of Equations (8) and (9), predicting the dv90 (as well as other characteristic diameters) as a function of the milling pressure, the feed rate, and the index b.

We confirmed the known inadequacy of the ductile–brittle transition models by Hagen and Kendall in predicting the smallest fragment size. We stress that deeper quantitative considerations on this point cannot be made on the basis of the presented data. Working with sub-micron size particles requires, in fact, the use of different particle size characterization methods (wet dispersion, Mie scattering) and the definition of a different set of milling trials, aimed at finding the limiting distribution of the different compounds, raising the pressure above 6 barg when necessary.

If these points are addressed and further investigated, we believe it will be possible to build a predictive model, enabling the estimation of the PSD of a milled product solely based on the mechanical properties measured via nano-indentation. Such a model of course remains valid only for the specific milling equipment used in the investigation; scaling the milling process up on larger mills or porting the process from one mill to another would require the construction of predictive models for each of them.

## Figures and Tables

**Table 1:** Measured Young modulus E and Hardness H ± their standard deviation. Comparison among different batches, different measurement sessions, and the literature data.

Material	Session	E (GPa)	H (Gpa)
Sodium Chloride	Literature	43.0–46.5 ± 1	0.213–0.44 ± 0.05
	Recrystallized	35.98 ± 3.0	0.23 ± 0.02
	Commercial	22.83 ± 4.3	0.20 ± 0.03
Tartaric Acid	Literature	43.34 ± 0.7	1.35 ± 0.05
	commercial 1	37.96 ± 7.2	1.09 ± 0.21
	commercial 2	32.18 ± 7.8	0.78 ± 0.18
	commercial 3	42.11 ± 7.3	0.85 ± 0.33
Lactose	Literature	21.44–23.7 ± 1	0.869–1.1 ± 0.06
	Recrystallized	27.59 ± 5.2	1.04 ± 0.15
	commercial 1	23.38 ± 5.4	0.79 ± 0.3
	commercial 2	21.44 ± 3.1	0.60 ± 0.18
compound A	1	9.04 ± 0.9	0.24 ± 0.03
	2	8.71 ± 1.3	0.28 ± 0.04
	3	12.41 ± 2.1	0.42 ± 0.06
	4	12.41 ± 1.1	0.42 ± 0.06
	5	8.19 ± 3.1	0.31 ± 0.15
compound B	1	8.19 ± 0.6	0.38 ± 0.04
	2	6.83 ± 0.8	0.30 ± 0.03
	3	4.76 ± 0.5	0.30 ± 0.02
compound C	1	6.64 ± 0.4	0.28 ± 0.03
	2	7.21 ± 2.4	0.38 ± 0.1

**Table 2:** Measured crack length c, fracture toughness $K_{C}$, brittleness index b, and critical diameter $d_{c}$ ± their standard deviation. Comparison among different batches, different measurement sessions, and the literature data.

Material	Session	c (μm)	Kc (Mpa m^1/2^)	b (m^−1/2^ × 1000)	dc (μm)
Sodium Chloride	Literature	/	0.5 ± 0.07	0.426	70.35
	Recrystallized	0	/	0	/
	Commercial	0	/	0	/
Tartaric Acid	Literature	/	0.17 ± 0.01	8.11	0.46
	commercial 3	9.5 ± 2.1	0.18 ± 0.07	9.10	0.6
Lactose	Literature	/	0.09 ± 0.03	9.55	0.26
	commercial 2	11.88 ± 1.48	0.14 ± 0.02	5.92	0.94
compound A	1	16.41 ± 6.32	0.11 ± 0.08	3.60	9.6
	2	21.50 ± 5.88	0.05 ± 0.02	6.52	1.47
	3	13.13 ± n.a.	0.035 ± n.a.	11.09	0.24
	5	17.15 ± 3.27	0.027 ± 0.006	16.08	0.12
compound B	1	5.5 ± 0.61	0.030 ± 0.004	13.85	0.17
	2	10.71 ± 2.96	0.024 ± 0.005	13.09	0.22
	3	7.5 ± 2.02	0.03 ± 0.006	11.90	0.28
compound C	2	16.15 ± 2.93	0.025 ± 0.004	17.64	0.09

## References

[1] Colombo P., Traini D., Buttini F. (2013). Inhalation Drug Delivery: Techniques and Products.

[2] Hickey A.J. (2004). Pharmaceutical Inhalation Aerosol Technology.

[3] Naik S., Chaudhuri B. (2015). Quantifying Dry Milling in Pharmaceutical Processing: A Review on Experimental and Modeling Approaches. J. Pharm. Sci..

[4] MacDonald R. (2017). Optimisation and Modelling of the Spiral Jet Mill. Ph.D. Thesis.

[5] Dogbe S.C. (2016). Predictive Milling of Active Pharmaceutical Ingredients and Excipients. Ph.D. Thesis.

[6] Nakach M., Authelin J.-R., Corsini C., Gianola G. (2019). Jet Milling Industrialization of Sticky Active Pharmaceutical Ingredient Using Quality-by-Design Approach. Pharm. Dev. Technol..

[7] Byard S.J., Tappertzhofen C., Andert D., Petzoldt C., Feth M.P., Bley O., Baumgartner B., Nagel N. (2013). An Example of How to Handle Amorphous Fractions in API during Early Pharmaceutical Development: SAR114137—A Successful Approach. Eur. J. Pharm. Biopharm..

[8] Choi W.S., Kim H., Kwak S.S., Chung H.Y., Chung H.Y., Yamamoto K., Oguchi T., Tozuka Y., Yonemochi E., Terada K. (2004). Amorphous Ultrafine Particle Preparation for Improvement of Bioavailability of Insoluble Drugs: Grinding Characteristics of Fine Grinding Mills. Int. J. Miner. Process..

[9] Storey R.A., Ymen I. (2011). Solid State Characterization of Pharmaceuticals.

[10] Alderborn G., Nyström C. (1996). Pharmaceutical Powder Compaction Technology.

[11] Levy A., Kalman H. (2001). Handbook of Conveying and Handling of Particulate Solids.

[12] Rantanen J., Khinast J. (2015). The Future of Pharmaceutical Manufacturing Sciences. J. Pharm. Sci..

[13] Pandey P., Bharadwaj R. (2017). Predictive Modeling of Pharmaceutical Unit Operations.

[14] Benassi A., Cottini C. (2021). Numerical simulations for inhalation product development: Achievements and current limitations. ONdrugDelivery.

[15] Bnà S., Ponzini R., Cestari M., Cavazzoni C., Cottini C., Benassi A. (2020). Investigation of Particle Dynamics and Classification Mechanism in a Spiral Jet Mill through Computational Fluid Dynamics and Discrete Element Methods. Powder Technol..

[16] Bhonsale S., Scott L., Ghadiri M., Van Impe J. (2021). Numerical Simulation of Particle Dynamics in a Spiral Jet Mill via Coupled Cfd-Dem. Pharmaceutics.

[17] Rodnianski V., Levy A., Kalman H. (2019). A New Method for Simulation of Comminution Process in Jet Mills. Powder Technol..

[18] Brosh T., Kalman H., Levy A. (2011). DEM Simulation of Particle Attrition in Dilute-Phase Pneumatic Conveying. Granul. Matter.

[19] Rozenblat Y., Grant E., Levy A., Kalman H. (2012). Selection and Breakage Functions of Particles under Impact Loads. Cheical Eng. Sci..

[20] Meier M., John E., Wieckhusen D., Wirth W., Peukert W. (2009). Influence of Mechanical Properties on Impact Fracture: Prediction of the Milling Behaviour of Pharmaceutical Powders by Nanoindentation. Powder Technol..

[21] Vogel L., Peukert W. (2003). Breakage Behaviour of Different Materials-Construction of a Mastercurve for the Breakage Probability. Powder Technol..

[22] Egart M., Janković B., Srčič S. (2016). Application of Instrumented Nanoindentation in Preformulation Studies of Pharmaceutical Active Ingredients and Excipients. Acta Pharm..

[23] Beake B.D., Harris A.J., Liskiewicz T.W., Ranganathan N. (2016). Advanced Nanomechanical Test Techniques. Materials Characterization: Modern Methods and Applications.

[24] Broitman E. (2017). Indentation Hardness Measurements at Macro-, Micro-, and Nanoscale: A Critical Overview. Tribol. Lett..

[25] Pérez Gago A., Kleinebudde P. (2017). MCC–Mannitol Mixtures after Roll Compaction/Dry Granulation: Percolation Thresholds for Ribbon Microhardness and Granule Size Distribution. Pharm. Dev. Technol..

[26] Duncan-Hewitt W.C., Weatherly G.C. (1989). Evaluating the Hardness, Young’s Modulus and Fracture Toughness of Some Pharmaceutical Crystals Using Microindentation Techniques. J. Mater. Sci. Lett..

[27] Shariare M.H., Leusen F.J.J., De Matas M., York P., Anwar J. (2012). Prediction of the Mechanical Behaviour of Crystalline Solids. Pharm. Res..

[28] Taylor L.J., Papadopoulos D.G., Dunn P.J., Bentham A.C. (2004). Mechanical Characterisation of Powders Using Nanoindentation. Powder Technol..

[29] Taylor L.J., Papadopoulos D.G., Dunn P.J., Bentham A.C., Dawson N.J., Mitchell J.C., Snowden M.J. (2004). Predictive Milling of Pharmaceutical Materials Using Nanoindentation of Single Crystals. Org. Process. Res. Dev..

[30] Zügner S., Marquardt K., Zimmermann I. (2006). Influence of Nanomechanical Crystal Properties on the Comminution Process of Particulate Solids in Spiral Jet Mills. Eur. J. Pharm. Biopharm..

[31] De Vegt O., Vromans H., Den Toonder J., van der Voort Maarschalk K. (2009). Influence of Flaws and Crystal Properties on Particle Fracture in a Jet Mill. Powder Technol..

[32] De Vegt M. (2007). Jet Milling from a Particle Perspective.

[33] Holdich R. (2020). Fundamentals of Particle Technology.

[34] Oliver W.C., Pharr G.M. (2004). Measurement of Hardness and Elastic Modulus by Instrumented Indentation: Advances in Understanding and Refinements to Methodology. J. Mater. Res..

[35] Chen J. (2012). Indentation-Based Methods to Assess Fracture Toughness for Thin Coatings. J. Phys. D. Appl. Phys..

[36] Roberts R.J. (1991). The Elasticity, Ductility and Fracture Toughness of Pharmaceutical Powders. Ph.D. Thesis.

[37] Knieke C. (2012). Fracture at the Nanoscale and the Limit of Grinding.

[38] Ghadiri M., Masuda H., Higashitani K., Yoshida H. (2006). Particle Impact Breakage. Powder Technology Handbook.

[39] Kendall K. (1978). The Impossibility of Comminuting Small Particles by Compression. Nature.

[40] Hagan J.T. (1979). Micromechanics of Crack Nucleation during Indentations. J. Mater. Sci..

[41] Müller F., Polke R., Schädel G. (1996). Spiral Jet Mills: Hold up and Scale Up. Int. J. Miner. Process..

[42] Cocco R., Arrington Y., Hays R., Findlay J., Karri S.B.R., Knowlton T.M. (2010). Jet Cup Attrition Testing. Powder Technol..

[43] Ghadiri M., Zhang Z. (2002). Impact Attrition of Particulate Solids. Part 1: A Theoretical Model of Chipping. Chem. Eng. Sci..

[44] Zhang Z., Ghadiri M. (2002). Impact Attrition of Particulate Solids. Part 2: Experimental Work. Chem. Eng. Sci..

